# COVID-19 disease and vaccination in pregnancy: understanding knowledge, perceptions and experiences among pregnant women and community leaders in Uganda

**DOI:** 10.1093/trstmh/trad028

**Published:** 2023-05-03

**Authors:** Phiona Nalubega, Ritah Namugumya, Flavia Zalwango, Agnes Ssali, Robert Mboizi, Lauren Hookham, Janet Seeley, Kirsty Le Doare

**Affiliations:** Medical Research Council/Uganda Virus Research Institute, and London School of Hygiene and Tropical Medicine Uganda Research Unit, P.O. Box 49, Entebbe, Uganda; Medical Research Council/Uganda Virus Research Institute, and London School of Hygiene and Tropical Medicine Uganda Research Unit, P.O. Box 49, Entebbe, Uganda; Medical Research Council/Uganda Virus Research Institute, and London School of Hygiene and Tropical Medicine Uganda Research Unit, P.O. Box 49, Entebbe, Uganda; Medical Research Council/Uganda Virus Research Institute, and London School of Hygiene and Tropical Medicine Uganda Research Unit, P.O. Box 49, Entebbe, Uganda; Makerere University–Johns Hopkins University Research Collaboration, P.O. Box 23491, Kampala, Uganda; St. George's, University of London, Cranmer Terrace, London SW17 0RE, UK; Medical Research Council/Uganda Virus Research Institute, and London School of Hygiene and Tropical Medicine Uganda Research Unit, P.O. Box 49, Entebbe, Uganda; London School of Hygiene and Tropical Medicine, 15-17 Tavistock Place, London, UK; Medical Research Council/Uganda Virus Research Institute, and London School of Hygiene and Tropical Medicine Uganda Research Unit, P.O. Box 49, Entebbe, Uganda; St. George's, University of London, Cranmer Terrace, London SW17 0RE, UK

**Keywords:** pregnancy, policy compliance, refusal to participate, vaccines

## Abstract

**Background:**

We investigated pregnant women and community leaders’ knowledge, perceptions and experiences of the coronavirus disease 2019 (COVID-19) vaccination program during pregnancy in Uganda and how this changed over the course of the pandemic.

**Methods:**

We conducted 20 in-depth interviews (IDIs) and two group discussions (GDs) with pregnant women and four GDs with community leaders in Kawempe division of Kampala, Uganda. The first round of IDIs/GDs were carried out in March 2021. In July 2021, telephone IDIs were conducted with 7 pregnant women and 10 community leaders randomly selected from first-round interview participants. Themes were analysed deductively drawing codes from the topic guides.

**Results:**

In the first round, the majority of participants thought COVID-19 was not real because of misconceptions around government messaging/motivation and beliefs that Africans would not be affected. In the second round, participants recognised COVID-19 disease, because of rising case numbers and fatalities. There was increased awareness of the benefits of the vaccine. However, pregnant women remained unsure of vaccine safety and quality, citing side effects like fevers and general body weakness. Role models and coherent public health messaging and healthcare workers were key enablers of vaccine uptake.

**Conclusions:**

Targeted and sustained COVID-19 communication and engagement strategies are needed, especially for pregnant women and others in their communities, to improve vaccine confidence during outbreaks.

## Introduction

The Coronavirus disease 2019 (COVID-19) pandemic, with its accompanying restrictions on movement and association, has dominated everyday life in many countries globally. Studies investigating the impact of COVID-19 infections in pregnancy have revealed that an infection is associated with increased risks of severe maternal and foetal complications, including maternal admissions to intensive care units and the risk for mechanical ventilation, comorbidities such as pre-eclampsia and thrombosis, maternal mortality and preterm and stillbirth.^1–3^

In December 2020, the vaccination of pregnant women became a priority to reduce disease burden in women and infants globally. Recommendations and possibilities for vaccination in this group have been inconsistent across countries and advice on safety in pregnancy changed during the course of the pandemic. However, several systematic reviews and pregnancy databases have not identified any obvious safety signals.^4^

In Uganda, the COVID-19 vaccine rollout was started with support from the COVAX initiative, which provided vaccines free of charge to African countries in March 2021.^5^ The vaccine rollout in Uganda began after the first 864 000 doses of AstraZeneca vaccine were received on 6 March 2021. By 8 October 2022, 25 783 131 people had been vaccinated.^6,7^ The first priority for vaccinations was healthcare workers (HCWs), security personnel, teachers, humanitarian frontline workers, individuals >50 y of age and those with underlying health conditions.^8,9^ Pregnancy was initially a contraindication for vaccination, with guidelines for COVID-19 vaccination in pregnant women being implemented after international guidelines started to appear that vaccines were safe in pregnancy.^10^ Messaging from the Ministry of Health on COVID-19 was provided on billboards, radio and social media, first on the disease and later on vaccination.

There is a paucity of literature about pregnant women's perceptions of new diseases, especially in the Ugandan context. However, some studies conducted among HCWs demonstrated that about 7 in 10 had sufficient knowledge about COVID-19 at the height of the pandemic, highlighting gaps in knowledge, even among frontline staff.^11^

As Opara^12^ observes, women in the USA are particularly suspectable to changes in health policy because they often access care for themselves and their children. Women in Uganda are also often the members of the family with the most frequent contact with healthcare providers. The Women's Manifesto 2021–2026^13^ recognizes the fact that the government of Uganda has put in place systems and structures to ensure efficient and effective delivery of health services. These include physical infrastructure facilities at all seven levels of service provision ranging from Community Health Worker Health Centre II (parish), Health Centre III (subcounty), Health Centre IV (county), district hospitals, regional referral hospitals and national referral hospitals. Uganda is reputed to have one of the best health policies as enshrined in the National Health Policy 2010, where service delivery is based on the Minimum Healthcare Package. The essence is provision of integrated healthcare at the subcounty level. Despite this, the health service delivery system faces multifaceted challenges leading to poor health indicators, particularly for women, manifesting in high maternal mortality rates (336/100 000 live births),^14^ infant mortality rates estimated at 31/1000 live births and 26% of the mothers still delivering outside formal health facilities.^15^ The budget allocation for health in Uganda remains <15% of the national budget recommended following the Abuja Declaration, which was agreed on by all African countries that are party to the declaration, including Uganda. The results include drug stockouts, lack of appropriate diagnostic equipment and poor attitudes of HCWs, largely resulting from their (HCWs) poor remuneration. Abortion services are illegal, but maternal, child and sexual health services are provided free of charge. We have previously shown^16^ that the distance to the health facility plays a role in vaccine acceptance, as does a lack of hospital staff, poor HCW attitudes, the spread of conspiracy theories and fake news in the media.

For this article we assessed pregnant women and community leaders’ knowledge, perceptions and experiences of COVID-19 disease and vaccination in pregnancy and how this changed during subsequent COVID-19 waves in Kampala, Uganda.

## Methods

This study was conducted in Kawempe, the largest division in Kampala, Uganda's capital, with an estimated population of 338 665.^17^ In-depth interviews (IDIs) and group discussions (GDs) were conducted at Kawempe Hospital, one of the national referral facilities for labour, delivery and neonatal care in Kampala and the surrounding areas.

### Recruitment

The study team purposely selected women 17–24 y and >25 y of age from antenatal care lists at Kawempe Hospital and approached women attending for care each day. Thirty-six pregnant women at different stages of pregnancy (assessed following gestational age described in the antenatal notes via the last menstrual period and/or ultrasound scan) were recruited from the antenatal department of Kawempe Hospital in this manner. Thirty-two community leaders from four villages surrounding Kawempe Hospital were purposely identified and selected to include a mix of religious leaders, local council leaders, village health teams (VHTs), traditional birth attendants and women leaders. All interviews took place in a private setting away from the antenatal clinic that was acceptable for both participants and researchers. After providing the study information to participants and allowing them time to consider joining the study, each participant was asked to provide written consent or a thumb print for those who were unable to read and write. For the less-literate participants, peers and partners served as witnesses of the consenting process.

### Data collection

In round 1 (March 2021), we conducted 20 IDIs and 2 GDs with pregnant women (eight women in each GD) and 4 GDs with community leaders (eight in each GD). Interviews were conducted after obtaining full written informed consent, and these lasted between 60 and 120 minutes. Women who took part in GDs were divided into two groups: younger mothers (17–24 y) and more mature women (>25 y), to avoid mature mothers overpowering the young mothers during the discussion. Participants in the GDs for leaders included both men and women, reflecting the leadership structure within the community.

In round 2 (July 2021), telephone IDIs were conducted with 7 pregnant women/breastfeeding mothers and 10 community leaders randomly selected from those who participated in the first round of interviews, to study the changes in knowledge and perceptions towards COVID-19 disease and vaccines. We developed semistructured topic guides to obtain participants’ sociodemographic information, information on the participants’ knowledge of COVID-19 disease and vaccines and the perceived impact of the disease and vaccines on pregnancy. Interviews were conducted in English or Luganda (the main local language in the area) and the GDs and IDIs were audio recorded. Saturation was defined as no new themes emerging.

### Data management and analysis

Data were transcribed verbatim into Word (Microsoft, Redmond, WA, USA), and if conducted in Luganda, translated into English. Two research team members with training in qualitative research methodologies ensured that all the information was captured and transcripts were cross-checked against the audio recordings to ensure accuracy. This was done through listening to the audio while reviewing the transcript; review was done by a different interviewer than the one who conducted the interview.

Data were coded both manually into an Excel spreadsheet (Microsoft) and electronically using NVivo 12 (QSR International, Doncaster, VIC, Australia). An initial coding framework was developed after reading the first five transcripts and additional codes were derived using an inductive process. The research team then agreed upon the final coding framework and the remaining transcripts were coded. Data were analysed deductively, drawing codes from the topic guides and research objectives.

## Results

A total of 68 participants took part in this study: 32 community leaders, 32 pregnant women and 4 lactating women (Table 1).

**Table 1.** Participant sociodemographic characteristics

Characteristics	Pregnant women (n=36)	Community leaders (n=32)
**Age (years)**		
17–24	13	3
25–29	14	5
30–39	5	8
40–49	4	6
≥50		10
**Sex**		
Female	36	20
Male		12
**Occupation**		
Religious leader		8
Traditional birth attendant		4
Village health team		12
Local council leader		8
Business/trader	4	
Unemployed	13	
Craft worker/tailor	3	
Housewife	13	
Health professional	2	
Teacher	1	
**Education level**		
Primary	15	
Secondary	13	
Tertiary	8	
**Marital status**		
Married	36	
Single	1	

The following three key themes were identified during analysis: knowledge and perceptions of COVID-19, barriers to COVID-19 vaccination uptake and facilitators of COVID-19 vaccine uptake (Table 2).

**Table 2.** Summary of themes

Major theme	Subtheme
Knowledge and perceptions of COVID-19	1. Questioning the existence of COVID-19
	Similarities and differences
	In the first round of interviews (March 2021), pregnant women (17–24, ≥25 y) and community leaders had similar thoughts: • COVID-19 is an invented illness • An illness aimed to reduce African population • An invented illness for purposes of making money • An illness that only affects Europeans • An illness that affects mostly the rich In the second round of interviews all pregnant women and some community leaders changed their perceptions and started to believe that COVID-19 existed. However, some community leaders in the second round did not change their perceptions and still believed that COVID-19 was being used as a trick to get money from the Whites.
Barriers to COVID-19 vaccination uptake	1. Safety concerns for the woman and unborn baby
	Similarities and differences
	• Young mothers (17–24 y of age) and community leaders expressed more concerns related to vaccine safety to the unborn baby during all rounds of interviews, whereas older women were more concerned about their own health.
	2. Vaccine quality concerns
	Similarities and differences
	• All participants expressed worry about the quality of the vaccine • High-profile stakeholders receive better vaccines • Lack of trust in government pharmaceuticals
	3. Concerns about consenting before receiving the vaccine
	Similarities and differences
	• All participants mentioned that consenting before receiving a vaccine was not a normal practice • Consenting is an agreement to accepting to receive an unsafe vaccine
	4. Using local herbs
	Similarities and differences
	• Community leaders and older women thought local herbs work better than the vaccine, while young mothers did not express any view on local herbs.
Facilitators of COVID-19	1. Increase in disease and death cases
vaccine uptake	Similarities and differences
	• All participants reported this same facilitator for COVID-19 vaccine uptake
	2. Role of influencers
	Similarities and differences
	• Only pregnant women thought they could be motivated by other people to take the vaccine
	3. Increased sensitisation
	Similarities and differences
	• All participants mentioned that information about COVID-19 vaccines and side effects increased acceptance

### Knowledge and perceptions of COVID-19

In the first round of interviews in March 2021, most participants had limited knowledge of COVID-19 or COVID-19 vaccines. It is important to note that at this time, information about COVID-19 was mainly obtained from the spaces outside the hospital, because of the lockdown.

All participants in the first round of interviews expressed similar thoughts about COVID-19 disease, such as an invented illness, an illness aimed to reduce the African population or an illness that only affects Europeans and rich people.

*People say that [the President] is targeting some money from the ‘whites’, so that is why they report that there is COVID-19. People said that ‘whites’ sent some money to [the President] when the COVID-19 pandemic started.* (IDI, pregnant woman round 1)

As Uganda was only mildly affected by the pandemic at the time of the round 1 interviews, few participants had seen the disease, as compared with the early stages of the human immunodeficiency virus (HIV) pandemic, making it difficult to believe it existed.

*The reason as to why some people have failed to believe that COVID-19 is real is because most people in this community have never seen any COVID-19 patient. They have never seen a COVID-19 patient like they do with HIV/AIDS.* (GD, community leader, round 1)

Even if participants recognised that COVID-19 existed, they reported that COVID-19 only affected Europeans and Americans based on the high number of cases of COVID-19 in the West compared with Uganda. Similarly, COVID-19 was perceived to be an illness of the rich since most of the victims in Uganda and elsewhere were thought to be among the wealthier class.

*Most people say that COVID-19 doesn't kill the Africans, it only kills the white people*. (GD, community leader, round 1)

*The community members also usually say that COVID-19 is an illness for the rich people not the poor….Whenever they show the death cases on TV, they usually show the people who have money.* (IDI, pregnant woman, round 1)

During the second round of interviews, pregnant women's thoughts about COVID-19 changed and they expressed the belief that the disease existed, because of rising cases and fatalities.

*The truth is now everyone believes that COVID is real. It's because most of us have seen people in our communities dying of COVID; for example, you would hear that someone has lost their mother and that the caretaker is also suffering from COVID-19. Children from boarding schools also came back home with the virus. When you hear people dying of COVID-19 you also must fear, it's enough proof that COVID is real, and it kills!* (IDI, pregnant woman, round 2)

Unfortunately, even with high numbers of COVID-19 cases in Uganda, during the second round of interviews there were still community leader participants who reported that COVID-19 was being used as a trick to get money from ‘the whites’.

*Even in this second wave, I still believe that the government just wants to get money from the whites by putting the country under lockdown.* (GD, community leader, round 2)

### Policy compliance

In the first round of interviews, vaccine hesitancy among participants was high due to misconceptions around government messaging/motivation and beliefs that Africans would not be affected. For example, 17 of 20 pregnant women who participated in the IDIs and more than a half of the pregnant women and community leaders in the six GDs did not believe in the existence of COVID-19 and felt no vaccine was necessary.

However, during the second round of interviews, COVID-19 vaccine confidence increased due to the increasing number of reported cases and fatalities as well as increased awareness of the benefits of the vaccine. For example, all the women interviewed had changed their perception and had started to believe that COVID-19 existed and that the vaccine would benefit them, whereas only 3 of 10 community leaders interviewed reported the same perceptions as in the first round.

### Barriers to COVID-19 vaccination uptake

#### Safety concerns for the woman and unborn baby

Most pregnant women and community leaders feared that vaccines would potentially harm unborn babies or lead to side effects such as miscarriage, blood clots and death.

*Community members usually say that the vaccines have some side effects because they heard that in the countries where the vaccine was first introduced, it caused blood clots.* (GD, community leader, round 1)

*To be honest, I fear the vaccine because I hear when you take it you get fever and you never know what will happen next. [As] an expectant mother, I worry about the reaction to this vaccine when it reaches inside to my baby.* (IDI, pregnant woman, round 2)

Young mothers (17–24 y) and community leaders in both rounds of interviews expressed similar concerns related to vaccine safety, such as miscarriage, death of the baby and fear that the vaccine would affect the unborn baby, whereas older women who had health conditions such as diabetes and high blood pressure were worried the vaccine would affect their own health.

In the first round of interviews, community leaders and pregnant women expressed worry about the quality of the vaccine they would receive, because they thought that high-profile stakeholders receive better vaccines and they did not trust government pharmacies. However, in the second round of interviews, participants did not show any concern with the quality of the vaccine they would receive.

#### Vaccine quality concerns

In addition to vaccine safety, participants also reported that they feared receiving the COVID-19 vaccine because they believed that government officials received the right vaccines and the rest of the population in the communities would receive vaccines that were not useful.

*[People in the community] say that the first-class people (high-profile stakeholders) received the good COVID-19 vaccine and [the ordinary citizens] will receive the fake vaccines.* (GD, community leader, round 1)

Some participants expressed a lot of scepticism about receiving genuine support or products from the government or pharmaceutical companies. They wondered what the motive for providing the vaccine was.

*First, people lost trust in the current government and the people who manufacture the vaccines. They are tired because they think that some people do things with [ill] intentions. Secondly, people will accept those vaccines depending on the ruling government. This current government just introduces drugs without testing their efficacy.* (GD, community leader, round 1)

*People's rumours that the government wants to kill its people through COVID-19 vaccination is one reason they would not accept the COVID-19 vaccine.* (IDI, pregnant woman, round 2)

While all participants raised concerns about consenting before receiving the vaccine, as this is not normal practice for other vaccines they had received or their children had received in the first round, in the second round, no group reported any concern about providing written consent to receive a vaccine.

*Vaccination has been taking place from the time we were born and we used not to consent. I am wondering why the current vaccination [requires that] people have to consent before receiving it.* (GD, community leader, round 1)

They perceived consenting as an agreement accepting to receive an unsafe vaccine, and in case of any side effects, they would be the ones to face the consequences.

In both rounds, community leaders and older women (≥25 y) thought that local herbs worked better than the COVID-19 vaccine, something that was not reported by the younger women.

Participants mentioned that the use of local herbal remedies as opposed to modern medicines or vaccination would hinder them from receiving the COVID-19 vaccine.

*Besides those vaccines, people have also [turned to] local herbs which they believe work [better] than the vaccines. Some people are aware that they got COVID-19 and they are already cured [from] using local herbs. I know most people trust their local herbs more than the Western medicine.* (GD, community leader, round 1)

### Facilitators of COVID-19 vaccine uptake

Participants in all rounds expressed similar facilitators to COVID-19 vaccine uptake, such as the observed increase in the number of cases and deaths reported by Ministry of Health and the increase in sensitization over radio and television. The pregnant women thought they could be motivated after knowing or witnessing other people take the vaccine and remain healthy, with no reported side effects.

Most of the participants reported that the increase in the number of cases and deaths from COVID-19 motivated many people to get vaccinated, including pregnant women. This was mostly reported during the second round of interviews (July 2021) when COVID-19 cases and deaths in the country increased.

#### Role of influencers

Some pregnant women felt that people may be more likely to accept vaccination if they are actively motivated by significant others such as HCWs, older people and senior government officials, like members of parliament, who were among the first people to receive the vaccine in Uganda or were vaccinated at a highly publicised event.

*What has inspired some people to receive the COVID-19 vaccine [are the] health workers. The health workers inspire us so much since they are the ones who treat us.* (IDI, pregnant woman, round 1)

*Even the elder people inspired us to receive the vaccine.* (IDI, pregnant woman, round 1)

*I no longer fear the COVID-19 vaccine because I got encouraged when I saw the members of parliament receive the vaccine.* (GD, pregnant women, round 1)

Participants also noted that having other people share their success stories could motivate them to receive the COVID-19 vaccine.

*I would like to receive that vaccine because among the people who were vaccinated, there was none who died.* (IDI, pregnant woman, round 1)

Eventually, many people were sensitized by the HCWs, community leaders and Ministry of Health about the benefits of vaccines and this motivated them to receive the COVID-19 vaccine. Some participants mentioned that some pregnant women might accept to get vaccinated to protect themselves and their family from getting COVID-19 because of the targeted awareness.

### Information about COVID-19 vaccines and side effects

Almost all participants interviewed mentioned that they valued the HCW's advice about the benefits and dangers of COVID-19 vaccination and this would compel them to take up the vaccine or choose not to go for vaccination.

*Once I am sensitised about the dangers and benefits of vaccination, I can make a choice. Once I notice that the benefits outweigh the dangers, I can accept.* (IDI, pregnant woman, round 1)

*If you sensitise the people about the benefits of the vaccines, they might accept to get vaccinated. If you don't explain to the person the benefits of a vaccine, they can't accept to receive them.* (IDI, pregnant women, round 1)

Given the novelty of the global COVID-19 pandemic and its unrelenting impact on health and well-being, findings showed some participants still did not believe that COVID-19 was real, even in the second wave when case numbers were high. Some participants’ perceptions changed in the second wave, mainly due to increased awareness of the disease. They had also been sensitised about the benefits of the vaccine and believed that it could help them go back to their normal way of life.

## Discussion

We describe increasing COVID-19 vaccine confidence in pregnant women and community leaders in an urban Ugandan setting over two COVID-19 waves (March–July 2021), highlighting key barriers to uptake and knowledge gaps that need to be addressed if vaccine coverage is to be optimised.

Initially, many pregnant women and community leaders were unwilling to receive the COVID-19 vaccine. Our findings suggest that this was largely driven by negative perceptions of the pharmaceutical industry and concerns over vaccine safety and/or the source of the vaccine. A study from East Africa reported similar findings, highlighting a need for better and earlier public health messaging in future epidemics.^18^ Studies in Nigeria and Kenya also revealed that study participants did not believe that the disease existed in their country.^19^

Additionally, a study about misconceptions of COVID-19 among Ugandan men reported participants who perceived COVID-19 as an illness for the White race, as we also found in our population.^20^

Previous studies about healthcare utilization in low- and middle-income countries also highlighted factors such as negative perceptions of healthcare quality^21^ and mistrust in government as barriers to uptake and utilisation of health services such as vaccination. This also aligns with our study, where participants said that they did not clearly understand the motives of the government and pharmaceutical companies in providing COVID-19 vaccines.^22^

Concerns around vaccine safety are commonly cited as reasons for not taking up vaccination in pregnancy.^23^ This is consistent with our results and a survey conducted in Kenya that reported concerns around fear of side effects as the major reason for not receiving the vaccine. A cross-sectional study of three centres in the USA between August and December 2020 reported vaccine safety concerns for their pregnancy (82%) as the leading concern for COVID-19 vaccination among pregnant women, followed by concerns about their own health (68%), vaccine effectiveness (52%) and the belief that the vaccine was not needed (22%).^24^ Among this population, the most trusted sources of COVID-19 information were also health professionals (obstetrician/gynaecologist: 42%; family doctor or primary care doctor: 28%; Centers for Disease Control: 13%). This finding suggests that attention should be placed on building trust in vaccines that may need to be rapidly rolled out during public health emergencies.^25^ Additionally, reassurance of the capabilities of the regulatory bodies in ensuring safety and effectiveness should be emphasized.^26^ More research is needed to identify and design effective messaging campaigns to overcome vaccine hesitancy among populations in each setting.^27^

In our setting, the strongest driver of maternal vaccine acceptance was the recommendation of a healthcare professional. This is consistent with findings from studies in the USA reporting that primary care providers were among pregnant women's most trusted sources of information.^23^ A systematic review and meta-analysis showed that other important drivers of vaccine uptake among pregnant women are vaccine-specific factors and disease-related risk perceptions, including the belief that the vaccine would benefit the mother and foetus and not cause harm.^28^ However, there are no data to show the number of pregnant women vaccinated against COVID-19 in Uganda even with widespread vaccine availability.

We also found vaccine effectiveness and belief in conspiracy theories to be strongly linked to vaccine confidence. This is contrary to a systematic review of beliefs surrounding pandemic or seasonal influenza and increased vaccine uptake, but, as in the second round of our study, they found a link between perceived severity and risk of hospitalisation and death and increased willingness to vaccinate. This finding is similar to research that was conducted in the USA, where participants who perceived COVID-19 to be a severe risk tended to have personal health concerns and therefore reported taking protective measures for themselves.^29^

Our findings suggest an increased willingness to get vaccinated as the pandemic evolved, specifically with more international recommendations for vaccination in pregnancy.^30^ However, there is no guarantee that willingness will continue to increase and newly issued recommendations also have the potential to generate confusion and increase vaccine hesitancy and refusal if not communicated appropriately, as we saw in our cohort where community leaders and pregnant women were unsure of the advice regarding vaccination in pregnancy.

We found that gaps in knowledge about COVID-19 disease and vaccines were filled by information from social media. Also, seeing role models such as the elderly or public figures getting vaccinated was a powerful tool that improved vaccine confidence. This finding is consistent with a study conducted in the USA that reported social media has increasingly been used as a source of vaccination data and as a powerful communication tool to increase vaccination.^31^

### Limitations

Our study was conducted in an urban setting and may not be applicable to rural areas. Our findings relate to a group of women and their communities in Kampala, and although many of our findings are similar to other Ugandan studies, they may not be representative of situations outside the country.

## Conclusions

Our findings indicate that people's knowledge and perceptions towards vaccination against COVID-19 changed as the pandemic matured and people had experienced disease cases and deaths. This finding suggests a need for increased communication and engagement strategies as soon as case numbers start to increase. Policymakers need to increase the sensitization of the population to create awareness of the disease and build confidence in the vaccine, especially among populations with additional concerns, such as pregnant women. Furthermore, evidence-based research and integration of the prospective COVID-19 vaccine into the routine vaccination schedule would strengthen the health system, improve uptake of the COVID-19 vaccine, particularly in pregnancy, and improve the health of the people in Uganda.

## Data Availability

Data that underlie the results reported in this article, after de-identification will be available for researchers upon request from the corresponding author. To gain access, data requesters will need to sign a data access agreement.
